# The ALPPS procedure as a novel “liver-first” approach in treating liver metastases of colon cancer: the first experience in Greek Cypriot area

**DOI:** 10.1186/s12957-016-0827-3

**Published:** 2016-03-08

**Authors:** Athanasios Petrou, Demetrios Moris, Pantelis Kountourakis, Mohammad Fard-Aghaie, Kyriakos Neofytou, Evangelos Felekouras, Alexandros Papalampros

**Affiliations:** Nicosia Surgical Department, Division of Hepatobiliary Pancreatic Surgery, Nicosia General Hospital, Nicosia, Cyprus; 1st Department of Surgery, Medical School, Laikon General Hospital, University of Athens, Athens, Greece; Lerner Research Institute, Cleveland Clinic Foundation, Cleveland, OH USA; Bank of Cyprus Oncology Center, Nicosia, Cyprus; Department of General and Abdominal Surgery, Asklepios Hospital Barmbek, Hamburg, Germany; Anastasiou Gennadiou 56, 11474 Athens, Greece

**Keywords:** ALPPS, Liver first, Colon cancer, Colorectal liver metastases

## Abstract

**Background:**

Despite recent advances in multimodality and multidisciplinary treatment of colorectal liver metastases, many patients suffer from extensive bilobar disease, which prevents the performance of a single procedure due to an insufficient future liver remnant (FLR). We present a novel indication for associating liver partition and portal vein ligation for staged hepatectomy (ALPPS) as a “liver-first” approach when inadequate FLR was faced preoperatively, in a patient with extensive bilobar liver metastatic disease of colon cancer origin.

**Case presentation:**

A 51-year-old lady was referred to our center due to a stage IV colon cancer with extensive bilobar liver disease and synchronous colon obstruction. During the multidisciplinary tumor board, it was recommended to proceed first in a palliative loop colostomy (at the level of transverse colon) operation and afterwards to offer her palliative chemotherapy. After seven cycles of chemotherapy, the patient was re-evaluated by CT scans that revealed an excellent response (>30 %), but the metastatic liver disease was still considered inoperable. Moreover, with the completion of 12 cycles, the indicated restaging process showed further response. Subsequent to a thorough review by the multidisciplinary team, it was decided to proceed to the ALPPS procedure as a feasible means to perform extensive or bilobar liver resections, combined with a decreased risk of tumor progression in the interim.

**Conclusions:**

All in all, ALPPS can offer a feasible but surgically demanding liver-first approach with satisfactory short-term results in selected patients. Larger studies are mandatory to evaluate short- and long-term results of the procedure on survival, morbidity, and mortality.

## Background

Despite recent advances in multimodality and multidisciplinary treatment of colorectal liver metastases (CLM), many patients suffer from extensive bilobar disease, which prevents the performance of a single procedure due to an insufficient future liver remnant (FLR). The latter seems to be the most important deciding factor in planning for liver resection. Portal vein ligation (PVL) or embolization (PVE) are standard approaches to induce liver hypertrophy of the FLR prior to hepatectomy in primarily non-resectable liver tumors [1].

The associating liver partition and portal vein ligation for staged hepatectomy (ALPPS) procedure was recently developed as a feasible means to perform extended liver resections [2]. It produces rapid, enormous hypertrophy of the remnant, making previously unresectable lesions resectable. Indications for ALPPS include any extensive liver resection with inadequate FLR [3].

Recent data are equivocal about the application of this technique in CLM as a feasible, efficient, and safe alternative to two-stage hepatectomy in terms of morbidity, oncological outcomes, and disease-free survival. A recent study showed that ALPPS is not inferior to two-stage hepatectomy in terms of intermediate oncological outcomes as well as perioperative morbidity in patients with CLM [4]. A multi-center study though demonstrated higher perioperative morbidity rates in ALPPS group compared to the two-stage hepatectomy approach, claiming the latter as the standard approach for performing R0 resection in patients with advanced CLM and inadequate FLR [5].

There are emerging data of the incorporation of ALPPS technique in therapeutic strategy in patients with CLM in order to achieve a macroscopic disease-free state and lead the patient as soon as possible to the oncological path. An example of such technique is the simultaneous resection of the primary colonic tumor with step one of the ALPPS procedure that may be feasible, safe, and efficient [6].

We present a novel indication for ALPPS as a “liver-first” approach when inadequate FLR was faced preoperatively, in a young patient with an extensive liver metastatic disease due to a splenic flexure colon cancer.

## Case presentation

**Fig 1:**
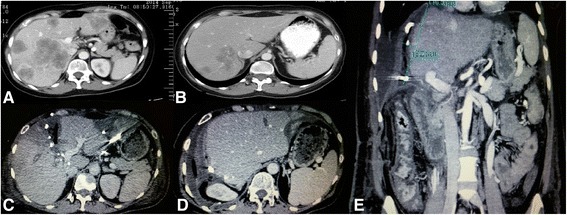
**a** Pre-chemotherapy abdominal CT scan showing multiple liver mestastases. **b** Post-chemotherapy abdominal CT scan showing response to treatment. **c**, **e** Abdominal CT scans showing adequate volume expansion after the step 1 of ALPPS procedure. **d** Postoperative abdominal CT scan without evidence of disease recurrence

A 51-year-old lady was referred to our center on 23/09/14 due to a stage IV colon cancer without any other major comorbidities. Her past medical history dated back almost 3 months ago with episodes of abdominal pain and constipation. Unfortunately, her previous investigation by colonoscopy showed an obstructing, infiltrative grade II adenocarcinoma in the area of splenic flexure, and the three-phase CT scans of the chest-abdomen-pelvis also revealed a stage IV disease with bilobar liver lesions (Fig. 1a).

During the multidisciplinary tumor board, it was recommended to proceed first in a palliative loop colostomy (at the level of transverse colon) operation and afterwards to offer her chemotherapy in the palliative setting. Due to the extensive nature of liver lesions, the disease was initially evaluated as unresectable.

Further investigation by mutation analysis of exons 2, 3, and 4 of the *KRAS* and *NRAS* and exon 15 of the *BRAF* gene revealed no mutations. Additionally, tumor markers’ evaluation revealed CEA = 1.341 ng/ml and CA 19.9 = 7.4 U/ml.

She was initiated on FOLFIRI [Irinotecan 180 mg/m^2^, folinic acid 175 mg, 5-fluorouracil (bolus) 400 mg/m^2^, 5-fluorouracil 2.400 mg/m^2^ (48 h pump)] plus cetuximab 500 mg/m^2^ on day 1, every 2 weeks via port-a-cath. Afterwards seven cycles of chemotherapy (3/10/14–13/01/15), the patient was restaged by full body CT scans performed by the standard three-phase protocol, that revealed an excellent response (>30 %), but liver lesions were again evaluated as unresectable. In addition, CEA evaluation showed a marked response (129, 2 ng/ml). Her restaging by CT chest-abdomen-pelvis scans, MRI liver (2/4/15) after the completion of 12 cycles of combined chemotherapy, as previously described, showed further response (>25 %) (Fig. 1b,c). Furthermore, dramatic decrease of CEA was also noted (6 ng/ml).

The decision to proceed to the ALPPS procedure as a feasible means to perform extensive or bilobar liver resections, combined with decreased risk of tumor progression in the interim, was made. The first step of the procedure including partitioning of the right lobe of the liver with right portal vein ligation was combined with four metastasectomies and two radiofrequency ablations (RFA) to the lesions of left lateral liver parenchyma—for 10 min at 45 W—as well as with closure of loop colostomy. A diverting loop ileostomy was performed instead in order to avoid major trauma infection. At this stage, a decision to avoid portal embolization was made since in that case, the possibility of inadequate future liver remnant was high due to the interventions in the left lateral liver and chemotherapy-induced liver injury.

In the tenth postoperative day (POD), a new abdominal CT scan was made that revealed adequate volume expansion of the future liver remnant and we decided to proceed to the second step of the procedure (Fig. 1d,e). CT volumetrics on POD 10 showed a total liver volume of 1690 cc with the left liver estimated to comprise approximately 40–50 % of the total volume.

The patient was discharged the POD 7 after the second step with an almost uneventful postoperative course except of a minor wound infection that was treated conservatively (wound dressing changes three times per week). On her first postoperative follow-up visit, her total bilirubin was 1.2 mg/dl and ALT/AST/ALP were 46/37/204 U/l. Of interest, her postoperative liver MRI showed no new lesions, without differences in the levels of CEA.

On 6/8/15 a left hemicolectomy was successfully performed. The histopathology report revealed an ypT3ypN2 (6+/14), gr II adenocarcinoma. Afterwards, the patient received three more months of combined chemotherapy (FOLFIRI + cetuximab, q2w, × six cycles) completed. The subsequent evaluation by CT chest-abdomen-pelvis scans by the end of December 2015 revealed no evidence of disease.

### Operative technique

**Fig 2:**
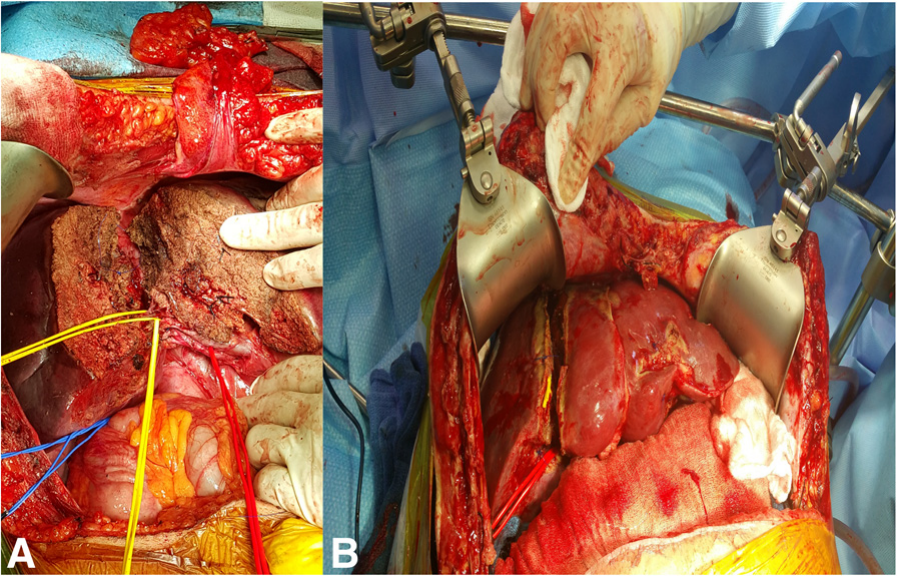
**a** Right hepatic artery and right hepatic duct kept into a vessel loop. **b** After in situ splitting, the right lobe was covered by a biomaterial

The operation was divided in two steps. The first consisted of exploratory laparotomy, assessment of resectability with intraoperative ultrasound, and positioning the tumor in relation to vessels. Four small lesions in left lateral lobe were resected. The liver was mobilized by dissecting the ligaments. The right liver lobe was completely mobilized from the cava vein. Hilar structures were exposed, and extensive nodal resection was performed. After the right portal vein branch being identified, it was divided. Right hepatic artery and right hepatic duct were identified and kept into a vessel loop (Fig. 2a). Biliary segment IV branch was also identified and kept into a vessel loop. After that, the hanging maneuver was performed and both right hepatic and a major short hepatic vein were also kept in a vessel loop to ensure better venous outflow. Finally, total parenchymal dissection over the hanging maneuver plane at the right of the falciform ligament was performed using bipolar coagulation irrigated with saline solution (CUSA, Harmonic, Aquamantys). After in situ splitting, the right lobe was covered by a biomaterial and the abdomen was drained and closed (Fig. 2b).

The second step of the procedure was completed by re-laparotomy. After careful adhesiolysis, the right hepatic artery, right hepatic duct, and the right hepatic vein were ligated. The liver resection was completed. The left lateral lobe was then fixed to the remnant falciform ligament. Finally, a drain was placed at the resection surface and the abdomen was closed. Since it was the very first experience of our team on the procedure and because we performed extensive metastasectomies and ablation to left lateral, we were conservative and kept the segment IV.

### Discussion

There is emerging literature on extending the indications for ALPPS as a rescue procedure when inadequate FLR is faced intraoperatively, during a simultaneous resection of colonic primary and liver metastasis [6] or after failure of PVL/PVE [1].

The current trend for the management of patients with synchronous colorectal cancer and liver metastatic disease is the so-called liver-first approach [7]. This modern policy has evolved as a result of the increasing evidence supporting preoperative chemoradiotherapy for rectal cancer as well as due to the view that it is the liver metastatic disease, rather than the primary cancer, that gives rise to systemic metastatic disease and defines the survival [7]. Following systemic chemotherapy, the liver resection is undertaken as the first operative intervention with the reasoning being that it is the burden of liver disease that is the likeliest course of subsequent metastasis [7]. Colorectal cancer resection is reserved as the second operative step, and for selected patients with rectal tumors (who have a complete endoscopic and radiologic response to chemoradiotherapy), there may then be the option to avoid pelvic surgery altogether [7].

ALPPS is a new two-stage hepatectomy that has partially evaluated postoperative outcomes in patients with malignant liver disease (primary and metastatic). It seems that patients with CLM are mostly benefited from this approach in terms of early postoperative outcomes [8]. It was recently shown that neoadjuvant chemotherapy significantly impairs hypertrophy of the FLR after ALPPS without impact on either morbidity or in-hospital mortality [9]. This is of great importance for the preoperative strategy especially in cases where ALPPS is used as a bail-out procedure.

As we have previously described, simultaneous right hemicolectomy and stage I ALPPS could be a feasible option [6].

All in all, our case stands for a description of a novel indication of ALPPS as a liver-first approach in a patient with cancer and extensive liver metastatic disease. To our knowledge, this is the first performance of the technique in the Greek Cypriot area with satisfactory results.

## Conclusions

All in all, ALPPS can offer a feasible but surgically demanding liver-first approach with satisfactory short-term results in selected patients. Larger studies are mandatory to evaluate short- and long-term results of the procedure on survival, morbidity, and mortality.

## Consent

Written informed consent was obtained from the patient for publication of this case report and any accompanying images. A copy of the written consent is available for review by the Editor-in-Chief of this journal.
